# Thermoelectric properties enhancement of p-type composite films using wood-based binder and mechanical pressing

**DOI:** 10.1038/s41598-019-44225-z

**Published:** 2019-05-27

**Authors:** Eunhwa Jang, Aswani Poosapati, Nathaniel Jang, Liangbing Hu, Michael Duffy, Marc Zupan, Deepa Madan

**Affiliations:** 1 Department of Mechanical Engineering, University of Maryland, Baltimore County, 1000 Hilltop Circle, Baltimore, MD 21250 USA; 2 Department of Materials Science and Engineering, University of Maryland, 4418 Stadium Drive, College Park, MD 20742 USA

**Keywords:** Physical sciences, Materials chemistry, Materials for devices

## Abstract

Thermoelectric generators (TEGs) fabricated using additive manufacturing methods are attractive because they offer the advantages of scalability, lower cost, and potentially higher power density than conventional TEGs. Additive manufacturing of TEGs requires active thermoelectric particles to be dispersed in a polymer binder to synthesize printable slurries, and printed films to be subsequently subjected to a long and high temperature curing to enhance their thermoelectic properties. A large amount of polymer binder present in composite films results in a sizable loss in the electrical conductivity. In addition, a long and high-temperature film curing results is a slow and energy intensive fabrication process. In this work, we demonstrate the feasibility of using a small amount (≤10^−3^ wt ratio) of novel nanofiber cellulose (NFC) as a binder to provide sufficient adhesion strength to hold the TE particles together in the composite films. We also demonstrate a pressure induced densification process to enhance the thermoelectic properties of printed composite films. This novel approach has the potential to fundamentally transform the manufacting method for printing TEGs by eliminating the need of long-duration and high-temperature curing. A higher applied pressure leads to a compact packing and densification of films resulting in an improvement in the electrical conductivity. The highest power factor achieved for best performing p-type thermoelectric-NFC composite film subjected to pressure induced densification is 611 μW/m-K^2^.

## Introduction

All energy conversion devices and processes (e.g., automobile engines, power transistors, light bulbs, industrial processes, etc.) are inefficient to a certain extent. During their operation, energy is often lost to the ysically in the form of heat. Energy harvesting devices, such as TEGs, can improve the energy efficiency by capturing part of this waste heat and converting it into electricity. TEGs utilize the waste heat in the form of temperature difference and directly convert it into useful electrical power^1^. Because TEGs can act as long-lasting power supplies, they eliminate the need for frequent battery replacements. In addition, these devices are reliable, largely maintenance free, and environmentally safe. These attractive features of TEGs make them ideal for use as power sources for sensors in hard-to-reach or remote locations. TEGs have become increasingly relevant due to the increasing need for power sources in inaccessible areas. The vast applications of TEGs include remote corrosion monitoring systems, implantable and wearable medical devices, structural health monitoring devices, radio frequency identification (RFID), and equipment monitoring, among others^2,3^.

The figure of merit (*ZT*) is a dimensionless quantity that determines the performance of the thermoelectric materials in a TEG device. *ZT* is defined by

$$ZT=\frac{{\alpha }^{2}\sigma }{k}T,$$

where *σ* is the electrical conductivity, *k* is the thermal conductivity, *T* is the temperature at which thermoelectric properties are measured, and *α* is the Seebeck coefficient. It is challenging to improve *ZT* due to the interdependent nature of *σ*, *k*, and *α*. TEGs are composed of alternately placed p-type and n-type thermoelectric elements. These elements are connected thermally in parallel but electrically in series. While TEGs are attractive as alternative power sources for various applications, these devices have some remaining challenges. Conventional TEGs built using pick-and-place methods are limited to low aspect-ratio elements. TEGs build using micro-fabrication technology have limited cost-effective scalability. Additive manufacturing methods (such as dispenser printing, screen printing, inkjet printing, stencil printing, etc.) can be used to address these limitations. A high aspect-ratio of the elements is desired in order to maintain the temperature difference across the device, which is necessary for generating a high voltage output. A high power-density can be obtained by packing a large number of elements in a small device^4,5^. Various research groups have evaluated additive manufacturing methods of printing TEG devices and have fabricated high-aspect-ratio thermoelectric elements^6,7^. The printed TEG elements can be made of polymers^8^, semi-metals^9^, or composites^10,11^. Both materials waste and manufacturing cost is reduced as a result of using printing technologies to fabricate TEGs. In addition, because a large number of high-aspect ratio elements can be packed in a small device, high voltage and high-power output can be produced.

Thermoelectric ink synthesis is a critical step in the printing of TEGs. Thermoelectric inks are typically synthesized by mixing active particles with a suitable insulating polymer binder and a solvent. The function of the solvent is to adjust the viscosity of the ink and that of the polymer binder is to hold the active particles together in the composite system. Various polymers have been explored for printing including a blend of panipol M resin, polyester and cellulose^7,12^, glass frit^12,13^, ethylene glycol^4^, polystyrene^13^, epoxy resin^10,14,15^, methocel, removable binders, 3-mercaptopropanoic acid (MPA)^16^. Madan *et al*. focused their research on use of epoxy resin as binders for inorganic TE particles based films and cured at high temperature. The highest ZT of 0.31 was achieved for mechanically alloyed (MA) n-type Bi_2_Te_3_^4,6,17,18^. The highest ZT of 0.41 was recorded for p-type Sb_2_Te_3_ printed composite films cured at 350 °C and ZT of 0.2 was recorded for MA Bi_0.5_Sb_1.5_Te_3_ with 8 wt. % extra Te films cured at 250 °C^6,10,18^. Navone *et al*. used polystyrene diluted in toluene as their binder to fabricate thermoelectric micro-modules on polyamide substrates with p and n-type pillars with 4 and 6 µWm^−1^K^−2^ power factors, respectively^5,13^. The higher mass loading of insulating polymer binder hindered the further improvement of electrical conductivity. Cho’s research group used removable binder with active TE particles for screen-printing the films. By removing the insulating binder, and tightly packing the active materials using glass frits this group was able to achieve the ZT of 0.61 for n-type Bi_2_Te_3_ screen printed films that were annealed at 500 °C for 15 mins^19,20^. Similarly Cho *et al*. achieved a ZT of 0.32 for p-type screen printed Sb_2_Te_3_ films by removing the binder and annealing at 500 °C^21^. Zhang *et al*. used wet chemical deposition of nanocrystalline Bi_2_Te_3_ and achieved ZT of 0.43^22^. Recently, Wang *et al*. used a very small amount of removable binder with n- and p-type Bi_2_Te_3_ based alloys and achieved a remarkably high ZT of 0.81 and 0.65 respectively^23^.

In addition to using insulating polymer as a binder to provide adhesion strength to thermoelectric particles for printable films researchers have also explored using doped conductive polymer as thermoelectric materials^24,25^. Pipe *et al*. reported PEDOT:PSS p-type polymer doped with DMSO achieved ZT of 0.42^26^. Researchers have also explored other conjugated polymers for TE purposes. Katz *et al*. used F4TCNQ to dope the P3HT polymer to improve the electrical conductivity^27,28^. Katz group also reported power factor of 0.63 μWm^−1^K^−2^ for 25 mol% TBAF-doped n-type ClBDPPV film^29^, and electrical conductivity of 0.004 Scm^−1^ for 75 wt.% Na-SG doped n-type NTCDI-2DT polymer film^30^. Muller group used PEO in addition to F4TCNQ to dope the P3HT polymer and achieved a power factor of 0.1 μWm^−1^K^−2^ ^31^. Some researchers have also used doped conductive polymer together with thermoelectric particles. Kato *et al*. made thin films with Bi_0.4_Sb_1.6_Te_3_ particles with PEDOT: PSS polymer along with PAA and recorded a ZT of 0.2^18^. Later, Bae *et al*. enhanced the thermoelectric properties obtained with PEDOT: PSS conducting system by adding Te nanorods and using chemical treatment methods^19^.

Despite these efforts most printed films have lower ZT values compared to those made from commercial bulk processes because of use of high amount of insulating binders during preparation of films. Additionally, most TE printed films with thermoset polymers require high curing temperatures and long curing time to improve electrical conductivity. The focus of this work is to explore cellulose hydrogel as a binder which has the advantages of abundant availability, mechanical strength, biodegradabilty, biocompatibility, and chemical accessibility for modification^32,33^. Nanofiber cellulose (NFC) also entails the advantages of excellent flexibility and lightweight. In addition, a low concentration of NFCs (1–2%) in water forms a stable hydrogel^32^. NFC has been explored for advanced electronics, biomedical and energy applications such as in organic light-emitting diode (OLED) devices, light weight paper actuators, bioactive paper, biosensor on NFC based paper, separators, and electrodes in a battery but not for TE applications^32–34^.

In addition to exploring a new binder for TE applications, we also focus on improving the properties of the obtained films through mechanical pressing. Mechanical pressing procedure has been used previously as a post-annealing process to improve the density and electrical properties. The application of mechanical pressure reduces the number of pores generated during the printing process, increases density of the resultant film, and brings the particles in contact. This results in reducing the resistivity of the films thereby improving the electrical conductivity^35,36^. In this work, we demonstrate pressure-induced densification can be used to control the thermoelectric properties of these composites eliminating the need of long-duration and high-temperature curing. We exploit this phenomenon to improve the TE properties of NFC hydrogel based composite films.

## Results and Discussions

**Figure 1:**
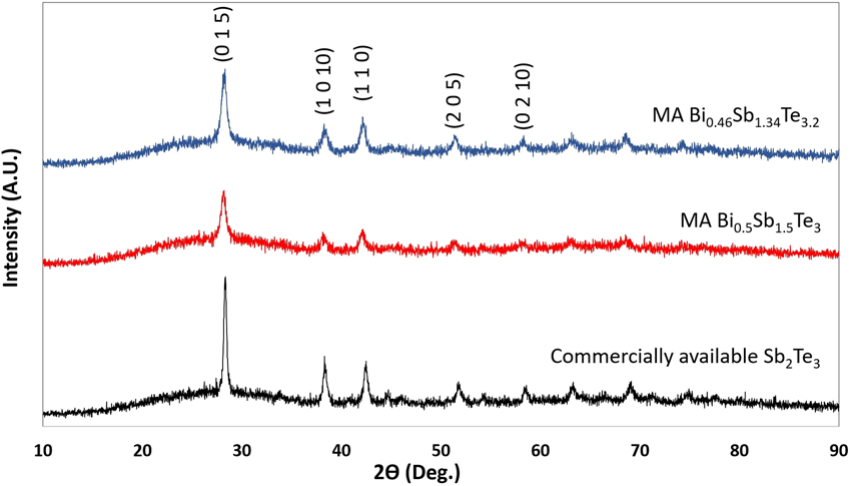
XRD patterns of Sb_2_Te_3_, Bi_0.5_Sb_1.5_Te_3_, and Bi_0.46_Sb_1.34_Te_3.2_ samples.

**Figure 2:**
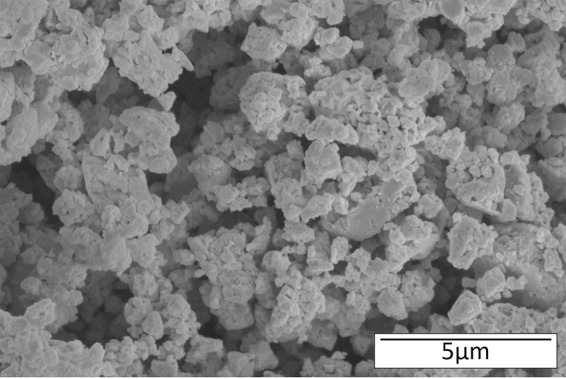
SEM image of the mechanically alloyed p-type particles.

X-ray diffraction (XRD) analysis was performed on commercially available Sb_2_Te_3_, MA Bi_0.5_Sb_1.5_Te_3_, and MA Bi_0.46_Sb_1.34_Te_3.2_ powder samples to compare the crystal structures. The XRD patterns of these samples are shown in Fig. 1. The XRD peaks conform to the standard pattern of Bi_0.5_Sb_1.5_Te_3_ (JCPDS49-1713). This observation confirms successful mechanical alloying in various Bi_0.5_Sb_1.5_Te_3_ samples. The structure of Bi_0.46_Sb_1.34_Te_3.2_ remains in the rhombohedral Bi_0.5_Sb_1.5_Te_3_ single phase^10^. Also, the addition of 8 wt% extra tellurium did not change the structure of Bi_0.46_Sb_1.34_Te_3.2_. Figure 2 shows the SEM image of MA p-type Bi_0.46_Sb_1.34_Te_3.2_ particles only, the particles size is in the range of 0.1~5 μm suitable for printing purposes.

**Figure 3:**
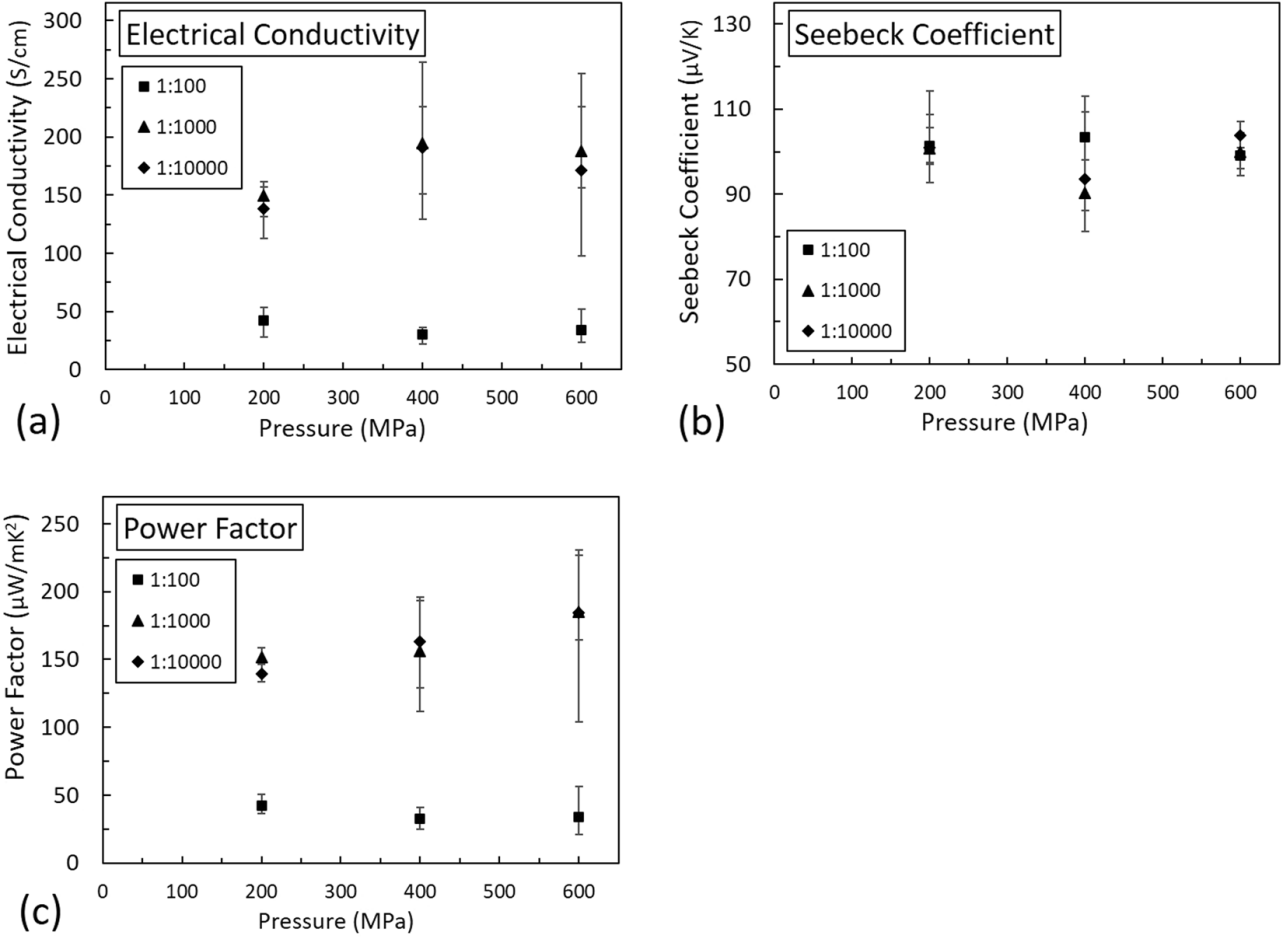
Effect of different pressure on the Sb_2_Te_3_ (p-type) NFC hydrogel based TE composite films: (**a**) electrical conductivity, (**b**) seebeck coefficient, (**c**) power factor.

TE composite films were prepared using various weight ratios (1:100, 1:1,000, and 1:10,000) of the NFC hydrogel binder and commercially available Sb_2_Te_3_ active particles on chitosan coated Kevlar substrate. Kevlar is a rough substrate therefore chitosan coating was used to achieve a smooth surface to deposit NFC hydrogel and TE particles composite films^23^. For 1:100 NFC hydrogel(binder) to Sb_2_Te_3_ particles wt ratio composite film, electrical conductivity values was 0.5 S/cm, Seebeck coefficient was 110 μV/K, and power factor was 0.6 μWm^−1^K^−2^. Subsequently, different samples were subjected to mechanical pressure of 200, 400, and 600 MPa. TE characterization were performed at room temperature. Figure 3 shows the TE properties after the mechanical pressing of Sb_2_Te_3_-NFC hydrogel composite films for various mass loading. An increase in the electrical conductivity was observed with the increase in the applied pressure for the 1:100 ratio samples. There was significant increase in electrical conductivity by varying the pressure from 0 MPa at 600 MPa, as shown in Fig. 3(a). The highest electrical conductivity was obtained for 1:1000 wt ratio of binder to TE particles at 600 MPa. No significant changes in the TE properties were observed for the applied pressure beyond 600 MPa. The electrical conductivity also improved as a result of increasing the wt% of active particles. The electrical conductivity increased significantly when binder to particles ratio was increased from 1:100 to 1:1,000. This increase is due to the lower amount of insulating binder in the 1:1,000 sample as compared to 1:100 sample. The difference in the electrical conductivity of 1:1,000 and 1:10,000 samples was not significant. We discuss the pressure-induced film densification and improvement in electrical conductivity using imaging analysis towards the end of this section.

Figure 3(b) shows the Seebeck coefficient value vs. applied pressure for various mass loading of hydrogel to Sb_2_Te_3_ particles. The positive value of the Seebeck coefficient confirms these are p-type particles and the Seebeck coefficient reduces with increase in the applied pressure for a given weight ratio. This observation is in agreement with findings from previous research studies that document inverse relationship between electrical conductivity and the Seebeck coefficient. Also, for a given applied pressure, a slight increase in the Seebeck coefficient is observed with the increase in the weight ratios of particles. This increase is due to more TE active particles compactly packed in the films. The power factor captures the effect of changes in both the electrical conductivity and the Seebeck coefficient. The best reported sample (1:1,000 weight ratio) has the highest electrical conductivity 233 Scm^−1^, Seebeck coefficient was 104 μV/K and power factor was 214 μWm^−1^K^−2^ at 600 MPa applied pressure.

**Figure 4:**
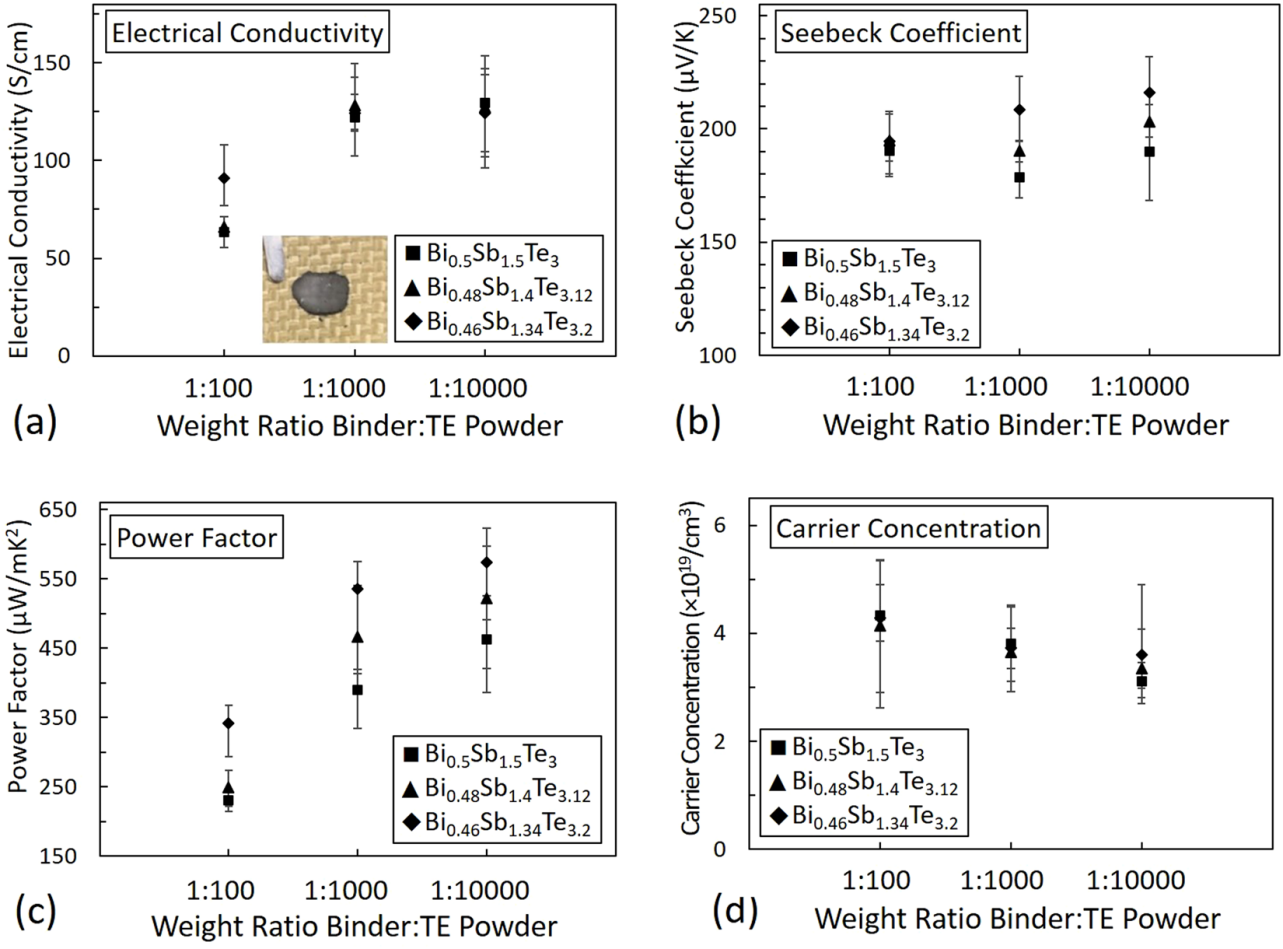
Effect of different NFC hydrogel to active particle weight ratios: (**a**) electrical conductivity, (**b**) Seebeck coefficient, and (**c**) power factor.

The above results demonstrate the successful use of NFC hydrogel as a binder for Sb_2_Te_3_ based thermoelectric films. In an effort to further improve the thermoelectric performance and achieve best possible results with NFC hydrogel binder, we utilize Mechanical alloy (MA) process to prepare thermoelectric particles by adding different concentrations of tellurium powder to Bi_0.5_Sb_1.5_Te_3_ to obtain Bi_0.48_Sb_1.4_Te_3.12_, and Bi_0.46_Sb_1.34_Te_3.2_. The next set of experiments were motivated by results from existing literature indicating an addition of extra tellurium to Bi_0.5_Sb_1.5_Te_3_ can significantly improve its thermoelectric properties^6,10,21,37^. Subsequently, we used these MA active particles together with NFC hydrogel binder to dropcast the films and their thermoelectric properties were characterized. Figure 4 shows thermoelectric properties for TE composite films with binder to active particle weight ratios of 1:100, 1:1,000 and 1:10,000. Bi_0.5_Sb_1.5_Te_3_, Bi_0.48_Sb_1.4_Te_3.12_, and Bi_0.46_Sb_1.34_Te_3.2_ were used as active particles. The image of TE composite film on Kevlar substrate is shown in Fig. 4(a). Because 600 MPa applied pressure yielded best TE results for composite Sb_2_Te_3_ films, the mechanical pressing for all variants of BiSbTe with NFC hydrogel was performed at this pressure. Figure 4a–d show the electrical conductivity, the Seebeck coefficient, power factor and, carrier concentration respectively. The results show the electrical conductivity increased with increase in binder to particle weight ratio for all the samples. The increase in the electrical conductivity was due to the reduced proportion of insulating binder in the film. The electrical conductivity value increased when binder to particle weight ratio changed from 1:100 to 1:1000 and 1:10,000 respectively. The sample with NFC hydrogel to Bi_0.46_Sb_1.34_Te_3.2_ ratio of 1:10,000 produced the best electrical conductivity value of 141 Scm^−1^.

There was no significant effect of added extra Te concentration on the electrical conductivity. The electrical conductivity for all the three composites were comparable for NFC hydrogel to particle weight ratios of 1:100, 1:1,000 and 1:10000. Mostly, an increase in the Seebeck coefficient was observed with an increase in the amount of added Te. Because Te is a high Seebeck coefficient metal, the Seebeck coefficient of the composite become higher when Te is added in higher concentrations. The best Seebeck coefficient (226 μVK^−1^) was observed for the sample with binder to Bi_0.46_Sb_1.34_Te_3.2_ ratio of 1:10,000. These high Seebeck coefficient values with extra Te are similar to our previous work of MA Bi_0.5_Sb_1.5_Te_3_ with addition of extra Te epoxy composite films^3,10,38^. The highest power factor of 611 μWm^−1^K^−2^ was obtained for the Bi_0.46_Sb_1.34_Te_3.2_ sample with binder to particle weight ratio of 1:10,000, with a corresponding electrical conductivity of 141 Scm^−1^ and a Seebeck coefficient of 226 μVK^−1^. The 8% extra Te films (i.e. the Bi_0.46_Sb_1.34_Te_3.2_ sample) resulted in the best power factor. Also, from Fig. 4d it can be observed that the films with higher amounts of Te had lower bulk carrier concentration values. Thus explaining the slight increase in Seebeck coefficient.

Also, the best sample among various MA samples was the 8% extra Te (Bi_0.46_Sb_1.34_Te_3.2_) sample with the binder to particle ratio of 1:10,000 as shown in Table 1. This sample achieved the power factor of about 611 μWm^−1^K^−2^. Overall, the power factor values increased with increase in the mass loading of particles and by addition of Te particles.

**Table 1:** Electrical conductivity, Seebeck coefficient, and power factor for composite NFC hydrogel TE films.

Thermoelectric powder	Wt ratio (binder: powder)	Applied pressure	Electrical conductivity	Seebeck coefficient	Power factor
Sb_2_Te_3_	1:1,000	600 MPa	233 S/cm	104 μV/K	214 μW/mK^2^
Bi_0.46_Sb_1.34_Te_3.2_	1:10,000	600 MPa	141 S/cm	226μV/K	611 μW/mK^2^

**Figure 5:**
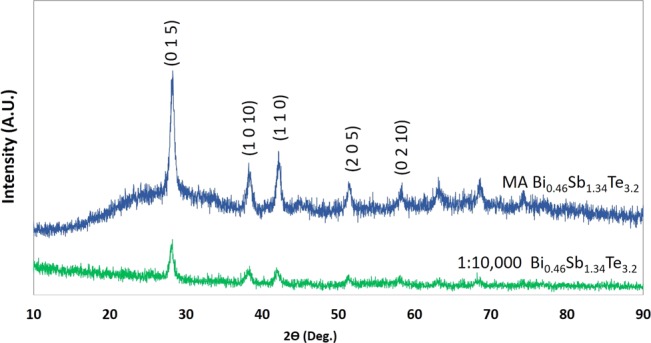
XRD patterns of pure MA and 1:10,000 Bi_0.46_Sb_1.34_Te_3.2_ samples.

To examine the effect of addition of hydrogel on particles, XRD analysis was performed for the 1:10,000 hydrogel_Bi_0.46_Sb_1.34_Te_3.2_ sample. This sample was chosen because it has the best power factor. The pressed sample was ground into powder using a mortor and pestel and then mounted into the sample holder for XRD. As shown in Fig. 5, the XRD analysis of the 1:10,000 hydrogel_Bi_0.46_Sb_1.34_Te_3.2_ sample shows evidence of amorphorization. This amorphization can be attributed to the mild sample oxidation and the presence of hydrogel in the composite system^11,39^. The reduction in the intensity of peaks can be attributed to the presence of hydrogel in the composite system.

In addition, we examined the potential degradation of film properties due to possible oxidation of film due to its exposure to ambient atmosphere. The data is reported in the Table 2 below. The electrical conductivity slightly increased with time and then become stable which implies that there is only slight oxidation due to extended exposure of the sample to the ambient atmosphere. (The data reported in the paper was collected immediately after sample preparation).

**Table 2:** Electrical conductivity, Seebeck coefficient measurement at various time interval for TE composite films (NFC hydrogel to Bi_0.46_Sb_1.34_Te_3.2_ in 1:10,000 wt ratio at 600 MPa).

Time	Electrical Conductivity	Seebeck coefficient
1hrs	107 S/cm	226 μV/K
12hrs	108 S/cm	220 μV/K
24 hrs	113 S/cm	220 μV/K
72 hrs	109 S/cm	224 μV/K
110 hrs	109 S/cm	220 μV/K

**Figure 6:**
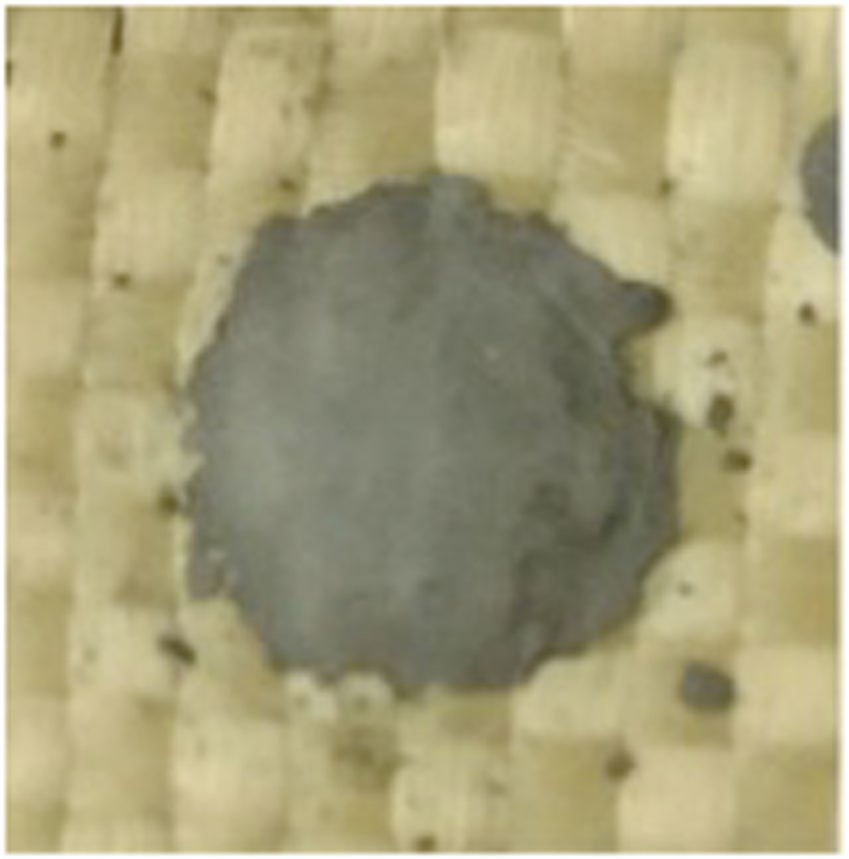
Images of TE composite films containing hydrogel and TE active particles after thermoelectric measurements.

Hydrogel is used in a small amount as a binder material for active thermoelectric particles. Because the thermoelectric properties of this composite are similar to those of other polymer-thermoelectric composites (such as epoxy-Bi_2_Te_3_ composite), we use equipment setup that are routinely used for polymer-thermoelectric composite materials. The role of the binder in the composite is to provide sufficient adhesion to hold the TE particles together. Because the composite films were uniform, they did not have visible cracks or flaws, and were not powdery (as shown the Fig. 6 in the manuscript) we concluded the NFC hydrogel provided sufficient adhesion to hold the TE particles together.

**Figure 7:**
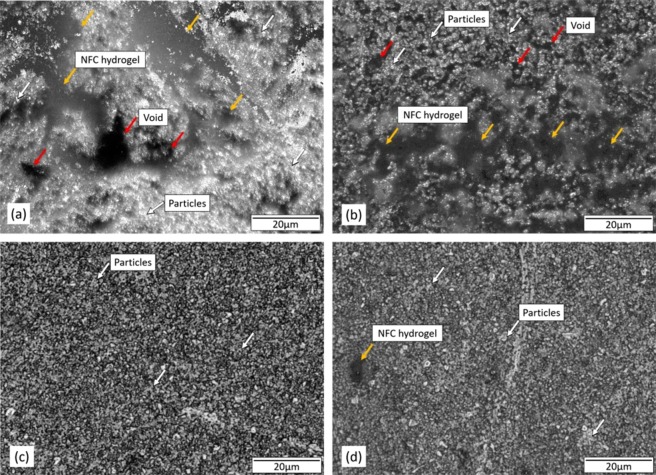
Optical microscopic images of samples with NFC hydrogel to MA Bi_0.46_Sb_1.34_Te_3.2_ weight ratio of (**a**) 1:100 and no applied pressure, (**b**) 1:1000 and no applied pressure, (**c**) 1:100 with 600 MPa of applied pressure, and (**d**) 1:1000 with 600 MPa of applied pressure.

To study the mechanism behind the improved results seen in thermoelectric characterizations, high-magnification optical microscope and SEM images of various films were captured. The images were analyzed for the information about the distribution and the packing of the TE active particles in the binder. Figure 6(a,b) show optical microscopic images of films with binder to MA Bi_0.46_Sb_1.34_Te_3.2_ weight ratio of 1:100 and 1:1,000, respectively. No mechanical pressure was applied for these two samples. These images show how the TE active particles are dispersed in hydrogel matrix. Yellow arrows show the NFC hydrogel binder and the red arrows indicate voids present in the TE composite films. A comparison of images shown in Fig. 7(a,b) reveals the films with binder to particle weight ratio of 1:1,000 has smaller amounts of NFC hydrogel binder phase than the sample with the weight ratio of 1:100. Also, the film, with binder to particle weight ratio of 1:100, has larger voids and pores than the film with the binder to particle ratio of 1:1,000. The presence of a large number of pores and voids limits the good contact between active TE particles. The electrical conductivity of TE hydrogel composite films depends on the electrical contacts between active TE particles. The electrical contact depends on the mass loading and the density of active particles. It is evident from the optical images that active particles are well connected and densely packed in samples with higher mass loading of particles. A higher density results in contact and less void space between active particles. Figure 7(c,d) show optical microscopic images of same samples as in 7(a) and (b) under 600 MPa applied mechanical pressure. As the applied pressure increased, active particles came close to the neighboring particles and started aggregating in hydrogel matrix. Additionally, particles and hydrogel interface is compressed and pores are filled with active particles resulting in better contact between particles. The outcome is a compact and dense film facilitating better electrical contact between grains of active particles. The contact between particles results in the formation of continuous path for charged carriers, which increases the electrical conductivity of the film^25,40–43^. It is clear from these optical microscope images that uniaxial cold pressing provides a way to reduce the pores and voids and improvise better electrical contact and aggregation between the particles.

**Figure 8:**
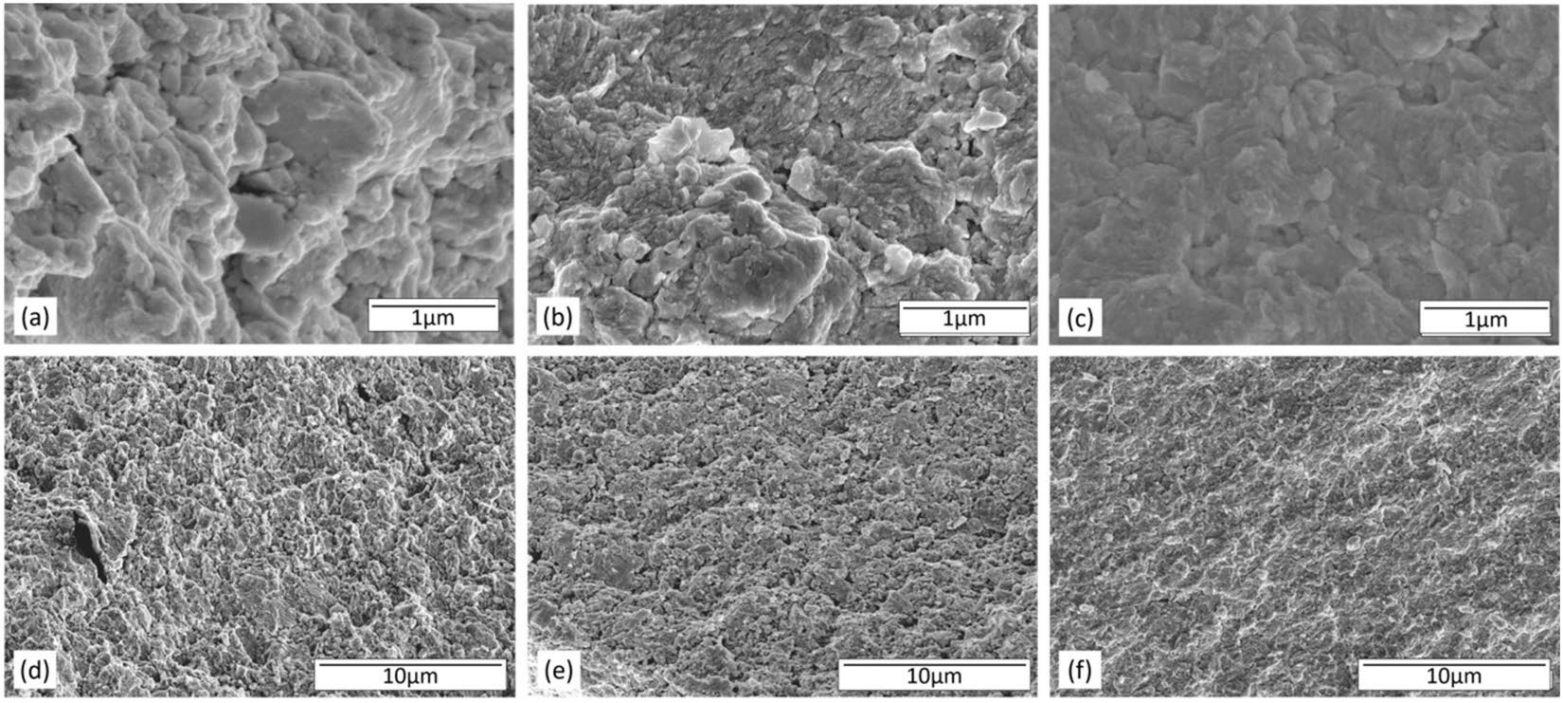
Cross sectional SEM images of pressed (**a**,**d**) 1:100 MA Bi_0.46_Sb_1.34_Te_3.2_ film, (**b**,**e**) 1:1000 MA Bi_0.46_Sb_1.34_Te_3.2_ film, (**c**,**f**) 1:10000 MA Bi_0.46_Sb_1.34_Te_3.2_ film.

To further confirm densification and better electrical contact phenomenon, SEM was performed on pressed films. Figure 8 shows the cross-sectional SEM images of pressed Bi_0.46_Sb_1.34_Te_3.2_ films at various mass loading. We picked Bi_0.46_Sb_1.34_Te_3.2_ films for the SEM analysis because it attained the best electrical conductivity and power factor values. Figure 8(a–c) correspond to the NFC binder to Bi_0.46_Sb_1.34_Te_3.2_ weight ratios of 1:100, 1:1,000, and 1:10,000, respectively, at 1 micron resolution. Figure 8(d–f) are SEM images of the same samples at 10 micron resolution. The high-resolution SEM images shown in Fig. 8(a,c) confirm that particles become tightly packed and well connected with each other when mass loading of particles was increased from 1:100 to 1:10,000. Also, the higher applied pressure reduced voids and caused neighboring particles to aggregate with each other resulting in better electrical contacts. SEM images confirm a higher mass loading and a higher applied pressure led to aggregation, better packing, and densification of films. As a result, better electrical contacts were formed and electrical conductivity improved.

**Figure 9:**
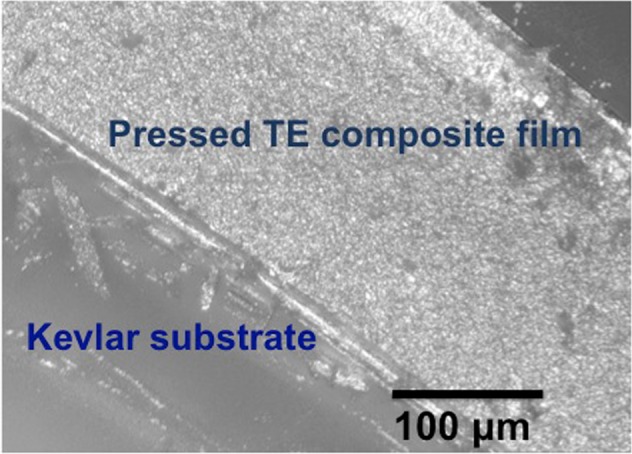
Side view image of pressed 1:10000 NFC Hydrogel and MA Bi_0.46_Sb_1.34_Te_3.2_ composite films pressed under 600 MPa on chitosan coated Kevlar films.

To understand the effect of high uniaxial pressure application on TE composite films deposited on chitosan coated Kevlar substrates, we performed high-resolution optical image microscopy as shown in Fig. 9. We chose a 1:10000 NFC hydrogel to Bi0.46Sb1.34Te3.2 TE composite films cured at room temperature and subjected them to an applied pressure of 600 MPa (for 5 minutes). The optical images of these TE composite films show no evidence of embedding of TE films in the Kevlar substrate even after 600 MPa applied pressure. Our hypothesis is that the chitosan coating on the Kevlar substrate provides a smooth surface and prevents the embedding and infiltration of TE films in Kevlar substrate. As a result, the thickness of the pressed TE composite films is reasonably uniform^23^.

## Conclusion

In summary, the NFC hydrogel was successfully used as a binder to drop-cast thermoelectric films. Unlike the need of high amount of binders in previous research for good adhesion of particles, a very small amount of novel nanofiber cellulose (NFC) (≤10^−3^ wt ratio) was sufficient to provide the needed adhesion strength to hold the TE particles together in the composite film. Unlike most other TE films with thermoset polymers and removable binders that require high curing temperatures and long curing time, NFC hydrogel TE composite films are cured at the room temperature in a short time to achieve good properties. Also, all samples were prepared in ambient atmosphere and dried at room temperature for 5 minutes and measured in ambient air. Therefore, the entire process of synthesizing inks, drop-casting and drying films, and pressure application can be completed within a couple of hours in ambient air. This novel manufacturing method eliminates the need of long durations for curing at high temperatures as performed by various researchers for either removing the binder or sintering the TE particles to improve TE properties. The best sample obtained using commercially available TE particles was the composite film in which the hydrogel to Sb_2_Te_3_ ratio was 1:1000 and a uniaxial pressure of 600 Mpa. This sample achieved the power factor of 214 μWm^−1^K^−2^. The best sample among various MA samples was the 8% extra Te (Bi_0.46_Sb_1.34_Te_3.2_) sample with the binder to particle ratio of 1:10,000. This sample achieved the electrical conductivity of 141 S/cm and the Seebeck coefficient of 226 μV/K which lead to the power factor of about 611 μWm^−1^K^−2^. Overall, as a result of adding extra Te to the MA Bi_0.5_Sb_1.5_Te_3_, using a high mass loading of TE particles in the binder, and the application of uniaxial pressure, a large increase in the power factor was achieved for TE composite films.

## Methods

### Mechanical alloying

Sb_2_Te_3_ (p-type) was purchased from the Santech Inc. and was used as TE active particles for ink preparation. Composite films were printed on flexible substrates using TE inks consisting of TE particles, a suitable binder, and a solvent. Previous research results indicate that with the addition of extra tellurium to Bi_0.5_Sb_1.5_Te_3_, the TE properties of printed composite films have improved^6,10,21^. Therefore, in this work we prepared three kinds of mechanically alloyed samples: Bi_0.5_Sb_1.5_Te_3_, MA Bi_0.48_Sb_1.4_Te_3.12_ (which consists 4% extra Te), and MA Bi_0.46_Sb_1.34_Te_3.2_ (which consists 8% extra Te) with the objective of improving the TE performance. For preparing the needed stoichiometric ratios, Bi (pellet, 99.999%, Santech Inc.), Sb (pellet, 99.999%, Santech Inc.) and Te (pellet, 99.999%, Santech Inc.) were mechanically alloyed in the nitrogen atmosphere. Each specific type of mixture, i.e. pellets amounting to Bi_0.5_Sb_1.5_Te_3_, Bi_0.48_Sb_1.4_Te_3.12_, and Bi_0.46_Sb_1.34_Te_3.2_, were placed in separate stainless steel jars. Stainless steel balls of 10 mm diameter were added in 1:15 pellets to balls ratio and the mixture was ball milled for 14 hours at 45 hz^10^.

### Ink synthesis

Empirical testing suggests the particle size of the active materials needs to be in the range from 1–10 μm for printing of good quality films. To produce the average particle size of 10 μm, wet grinding was performed on MA particles. A weight ratio of 1:1:10 of the resultant MA TE particle, isopropyl alcohol, and grinding balls were placed in stainless steel jars and ball milled at 30 hz for 150 minutes. The resulting fine powder was subsequently mixed with NFC hydrogel. The NFC hydrogel was obtained from Dr. Liangbing Hu’s research group. Inks of proper viscosity were prepared by mixing the fine MA TE particles with NFC hydrogel and appropriate amounts of distilled water. Various weight ratios of binder to particles are evaluated to attain the best possible TE properties of drop-casted TE films. The inks were thoroughly mixed using a vortex mixer (MX-S, Scilogex) for 5 minutes and were sonicated for 30 minutes using ultrasonic bath (CPS2800H, Emerson).

### Film fabrication

The inks were then drop-casted on chitosan coated Kevlar substrates (Kevlar substrate, Fibre Glast developments Corporation) and dried at room temperature for 5 minutes. Drop casted films were prepared by using various inks in different NFC hydrogel to particle (e.g., Sb_2_Te_3_, Bi_0.5_Sb_1.5_Te_3_, Bi_0.48_Sb_1.4_Te_3.12_, and Bi_0.46_Sb_1.34_Te_3.2_) weight ratios of 1:100, 1:1,000, and 1:10,000. The drop casted composite films were densified by applying mechanical pressure in the range of 10 MPa to 600 MPa for 5 minutes.

### Characterizations

Seebeck coefficient and electrical conductivity measurements were performed in ambient air using a custom-built setup. The electrical conductivity was measured using the van der Pauw method. The van der Pauw method measures the vertical and the horizontal resistance and calculates the sheet resistance for arbitrary and irregular shape sample^44^. To measure the vertical and the horizontal resistance, a current of 1000 µA was forced through the films and corresponding voltage drop were measured using the Keithley 2400 source meter. In order to reduce measurement errors, multiple readings of resistance were taken for each direction. The thickness of the pressed sample was measured using a micrometer (Mitutoyo). Substrate thickness (222 μm) was subtracted from the total thickness (421 μm) to find the film thickness (218 μm). In the table below, we show measurements for Kevlar substrate thickness, total thickness, film thickness, and area of one film. Sample thickness was measured at 4 different locations on the film and the film thickness variation is relatively small (<10 μm). Area of the pressed TE composite film is 35 mm^2^. The Kevlar substrate is coated with chitosan to make the rough Kevlar substrate smooth. This chitosan coating also helps in reducing the potential film embedding into the substrate. The electrical conductivity of films was obtained by calculating the reciprocal of the product of sheet resistance and thickness of each film. The Seebeck coefficient measurements were performed using a custom-built test setup to measure the voltage difference, ΔV, for a given temperature difference, ΔT. To reduce measurement errors, the system is enclosed in a thermally insulated case and protected from viable fluctuations of the ambient atmosphere. For measuring the Seebeck coefficient, a custom-built setup was utilized in which two peltiers were used to create a temperature difference across the sample by forcing voltage through the peltiers. Metal electrodes were made on top of the sample using Au paste for Seebeck and electrical conductivity measurements. The voltage and temperature probes on each of the ends were positioned close together to minimize the difference between actual and measured values. In order to further reduce the possibility of errors, multiple readings of ΔV were taken for each ΔT. The voltage difference was measured at six different values of ΔT (0 K, 1.6 K, 3.2 K, 4.8 K, 6.4 K, and 8.0 K). The Seebeck coefficient $({\rm{\alpha }}=\,\frac{{\rm{\Delta }}V}{{\rm{\Delta }}T})$ was calculated as the slope of the (ΔT, ΔV) line. Power factor was calculated using the equation

$${\rm{PF}}={{\rm{\sigma }}{\rm{\alpha }}}^{2},$$

where σ is the electrical conductivity and α is the Seebeck coefficient. The carrier concentration measurements were performed using ecopia-3000.
